# Effect of Niacin on Inflammation and Angiogenesis in a Murine Model of Ulcerative Colitis

**DOI:** 10.1038/s41598-017-07280-y

**Published:** 2017-08-02

**Authors:** Hesham Aly Salem, Walaa Wadie

**Affiliations:** Department of Pharmacology and Toxicology, Faculty of Pharmacy, Cairo University, Cairo, Egypt

## Abstract

Butyrate and niacin are produced by gut microbiota, however butyrate has received most attention for its effects on colonic health. The present study aimed at exploring the effect of niacin on experimental colitis as well as throwing some light on the ability of niacin to modulate angiogenesis which plays a crucial role of in the pathogenesis of inflammatory bowel disease. Rats were given niacin for 2 weeks. On day 8, colitis was induced by intrarectal administration of iodoacetamide. Rats were sacrificed on day 15 and colonic damage was assessed macroscopically and histologically. Colonic myeloperoxidase (MPO), tumour necrosis factor (TNF)-α, interleukin (IL)-10, vascular endothelial growth factor (VEGF), angiostatin and endostatin levels were determined. Niacin attenuated the severity of colitis as demonstrated by a decrease in weight loss, colonic wet weight and MPO activity. Iodoacetamide-induced rise in the colonic levels of TNF-α, VEGF, angiostatin and endostatin was reversed by niacin. Moreover, niacin normalized IL-10 level in colon. Mepenzolate bromide, a GPR109A receptor blocker, abolished the beneficial effects of niacin on body weight, colon wet weight as well as colonic levels of MPO and VEGF. Therefore, niacin was effective against iodoacetamide-induced colitis through ameliorating pathologic angiogenesis and inflammatory changes in a GPR109A-dependent manner.

## Introduction

Commensal microbiota in the gut have profound effects on human health^1, 2^. They promote colonic health through production of the short-chain fatty acids (SCFAs) by fermentation of dietary fiber. Among SCFAs, butyrate has received most attention for its effects on colonic health^3^. Previous studies proved that butyrate attenuated colonic inflammation and stimulated colonic repair^4–6^. Moreover, it improved the efficacy of mesalazine in experimental colitis models and inflammatory bowel disease (IBD) patients^7–9^. The cell-surface receptors identified for butyrate are GPR43 and GPR109A which is also known as hydroxycarboxylic acid receptor 2 (HCA2 or HCAR2) or niacin receptor 1 (NIACR1). These receptors are G-protein-coupled and are expressed in colonic epithelium, adipose tissue and immune cells^10, 11^. Singh *et al*. revealed that GPR109A signaling imposed anti-inflammatory properties in colonic antigen-presenting cells, which in turn induced differentiation of Treg cells and interleukin (IL)-10 producing T cells. GPR109A was also essential for the expression of IL-18 in colonic epithelium. *Niacr1* ^−/−^ mice showed enhanced susceptibility to colitis and colon cancer^12^.

Moreover, high intake of dietary fibre protected against dextran sulphate sodium (DSS)-induced colitis, an effect that was found to be a GPR109A dependent^13^.

The pharmacologic agonist for GPR109A is niacin (nicotinic acid) which is also produced by gut microbiota^10, 11^. Niacin, when taken in pharmacological doses, modifies lipid profile in circulation by acting as a GPR109A agonist in adipocytes. At these high doses, niacin is likely to reach the colon at concentrations high enough to exert GPR109A-dependent effects^12^. Niacin deficiency in humans results in pellagra, characterized by intestinal inflammation, diarrhea, dermatitis and dementia^14^. Singh and his colleagues demonstrated that niacin protected antibiotic-treated mice from weight loss, diarrhea, bleeding and colon cancer induced by administration of azoxymethane (AOM) and DSS^12^. However, the effect of niacin on experimental colitis model is no longer studied. The present study was, therefore, conducted to explore the effect of niacin on iodoacetamide-induced colitis and to throw some light on the ability of niacin to modulate angiogenesis which plays a crucial role in the pathogenesis of IBD^15–17^. The effect of niacin on the levels of both angiogenic and antiangiogenic factors was investigated in this study. Moreover, the role of GPR109A in mediating such beneficial effects of niacin was examined.

## Materials and Methods

### Materials

Niacin was provided from Nice chemicals Pvt. Ltd. (Kochi, Kerala, India). Iodoacetamide and mepenzolate bromide (MPN) were obtained from Sigma Chemicals Co. (St. Louis, MO, USA). Rat TNF-α ELISA kit was purchased from R&D Systems (GmbH, Wiesbaden, Germany). Rat IL-10 ELISA kit was from Elabscience Biotechnology Co., Ltd (Wuhan, Hubei, China). Rat angiostatin ELISA kit was from LifeSpan BioSciences, Inc. (Seattle, WA 98121, United States). Rat specific VEGF and endostatin ELISA kits were from Cusabio Biotech Co., Ltd. (Wuhan, Hubei, China).

### Animals

Adult male Wistar rats, weighing 150–200 g each, were obtained from the Modern Veterinary Office for Laboratory Animals (Giza, Egypt) and were left to acclimatize for one week before subjecting them to experimentation. They were provided with a standard pellet diet and given water *ad libitum*. The animals were kept at a temperature of 22 ± 3 °C and a 12-hour light/dark cycle as well as a constant relative humidity throughout the experimental period. The investigation complies with the *Guide for the Care and Use of Laboratory Animals* published by the US National Institutes of Health (NIH Publication no. 85–23, revised 2011) and was approved by the Ethical Committee for Animal Experimentation at Faculty of Pharmacy, Cairo University (Permit Number: PT 1835).

### Iodoacetamide-induced colitis

The rats that had been fasted for 24 hours but had free access to drinking tap water were lightly anesthetized with ether. Colitis was then induced by instillation of 0.1 ml of 4% iodoacetamide dissolved in 1% methylcellulose into the colon via a catheter placed 8 cm proximal to the anus.

### Experimental design

Rats were randomly assigned to four groups of eight animals each as follows: two control group; normal and colitis controls, and two niacin-treated groups (80 and 320 mg/kg). The drug/vehicle was administered orally once per day for 2 weeks. On day 8, colitis was induced in all groups except normal control which received 1% methylcellulose intrarectally. Animals were weighed just before iodoacetamide administration and just before autopsy.

Twenty-four hours after the last dose of treatment, the rats were sacrificed by cervical dislocation. The distal 10 cm of colon was excised, opened longitudinally, rinsed in ice-cold normal saline, cleaned of fat and mesentery, blotted on filter paper, and weighed. Colon wet weight (mg/g body weight) was calculated as a reflection of the severity of colitis. The colon segment was then cut longitudinally into two parts: one specimen was fixed in 10% formalin and preserved for histological examination, and the other was homogenized in ice-cold normal saline to obtain a 10% homogenate for assessment of the chosen biochemical parameters.

### Determination of biochemical parameters

The colon homogenate was divided into two aliquots. One aliquot was mixed with an equal volume of 100 mmol/L phosphate buffer pH 6 containing 1% hexadecyltrimethylammonium bromide. The mixture was freeze-thawed, sonicated for 10 seconds and centrifuged at 10000 rpm for 15 minutes at 4 °C. The supernatant was used for spectrophotometric estimation of myeloperoxidase (MPO) activity^18^. The second aliquot was used for assaying tumour necrosis factor (TNF)-α, IL-10, vascular endothelial growth factor (VEGF), angiostatin and endostatin using specific enzyme-linked immunosorbent assay (ELISA) kits.

### Histopathological assessment

Transverse sections, 4–6 μm in size, were prepared from paraffin-embedded colon segments from each animal. The sections were stained with hematoxylin and eosin (H&E) and examined under a light microscope. They were graded individually by a pathologist blinded to the treatment regimen. Each section was assigned a damage score between 0 and 3 for each of five parameters, namely; mucosal necrosis, mucosal inflammatory cells infiltration, sub-mucosal inflammatory cells infiltration, fibrosis and sub-mucosal oedema. The scores for the five parameters measured for each rat were summed to obtain the “total histology score”, being maximally 15 (three as the maximum for the five parameters examined). The data were then represented using a box plot.

### Role of GPR109A

To further characterize the role of the GPR109A receptor, animals were allocated into 6 groups, 5 rats each, and were treated as follows: normal and colitis controls (received the vehicle), two niacin-treated groups (80 and 320 mg/kg/day), and two niacin-treated groups (80 and 320 mg/kg/day) that were also given MPN, a GPR109A inhibitor, by intraperitoneal injection in a dose of 5 mg/kg/day^19, 20^. Same experimental design was repeated and the obtained colon samples were used for the biochemical assessment of both MPO and VEGF.

### Statistical analysis

All data obtained, except for histological scores, were expressed as means ± SEM and analyzed using one-way-analysis of variance test (one-way ANOVA) followed by Tukey’s Kramer multiple comparison test. Histological scores were presented as median and analyzed using Kruskal-Wallis test followed by Dunn’s test. Statistical analysis was performed using GraphPad Prism software, version 6.01 (GraphPad Software Inc., San Diego, CA). For all the statistical tests, the level of significance was set at *p* < 0.05.

## Results

### Body weight

**Figure 1:**
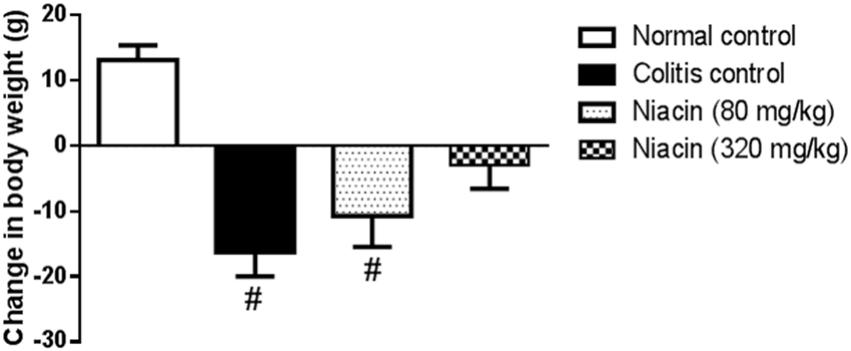
Effect of pretreatment with niacin on the increase in body weight of animals with iodoacetamide-induced colitis measured from the time of induction of colitis until sacrifice. Data are expressed as means ± SEM of 8 animals. ^#^ *P* ≤ 0.05 vs. normal control.

Iodoacetamide-induced colitis led to a decrease in body weight of rats (*p* < 0.0001). Pretreatment with niacin, especially, at the high dose level (320 mg/kg) tended to protect against such a decease in body weight (*p* = 0.1318) (Fig. 1).

### Colon wet weight

**Figure 2:**
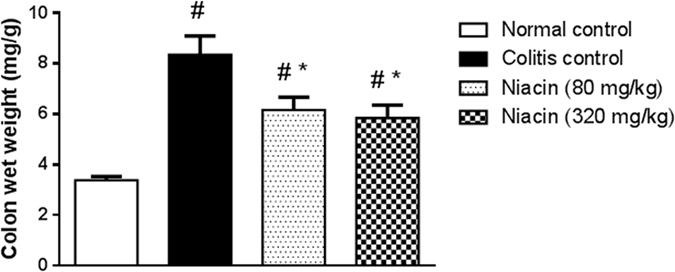
Effect of niacin on colon wet weight in rats with iodoacetamide-induced colitis. Data are expressed as means ± SEM of 8 animals. ^#^ *P* ≤ 0.05 vs. normal control, *P ≤ 0.05 vs. colitis control.

Intrarectal administration of iodoacetamide resulted in a 1.5-fold increase in colon wet weight as compared to the normal control group (3.39 ± 0.14 *vs*. 8.36 ± 0.73 mg/g). This obvious increase was prevented by pretreatment with niacin in a dose-dependent manner (Fig. 2).

### MPO, TNF-α and IL-10

**Figure 3:**
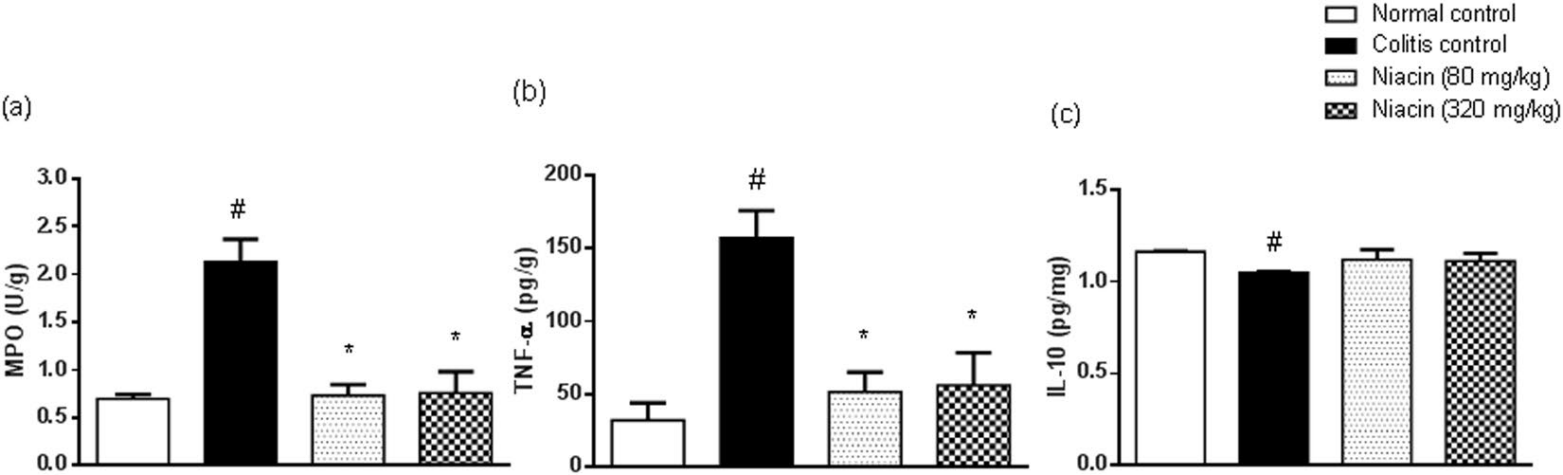
Effect of niacin on inflammatory and anti-inflammatory parameters in colonic tissues of rats with iodoacetamide-induced colitis. (**a**) Myeloperoxidase (MPO) activity, (**b**) tumour necrosis factor (TNF)-α levels, (**c**) IL-10 levels. Data are expressed as means ± SEM of 8 animals. ^#^ *P* ≤ 0.05 vs. normal control, *P ≤ 0.05 vs. colitis control.

Iodoacetamide caused spike increase in colonic MPO activity and TNF-α level. These derangements were largely prevented by pretreatment with niacin (Fig. 3a,b).

The colonic level of anti-inflammatory cytokine IL-10 was, however, reduced in colitis rats, an effect that tended to be prevented by niacin (Fig. 3c).

### VEGF, angiostatin and endostatin

**Figure 4:**
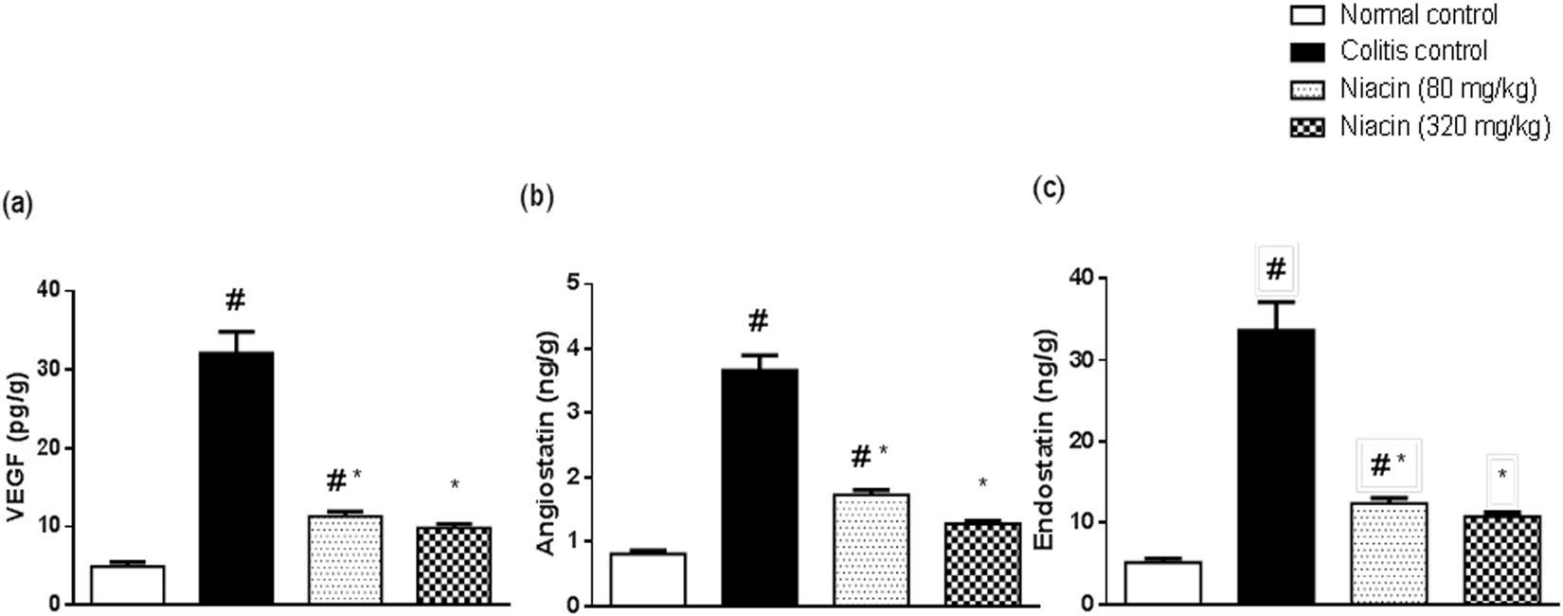
Effect of niacin on proangiogenic and antiangiogenic factors in colonic tissues of rats with iodoacetamide-induced colitis. **(a)** Vascular endothelial growth factor (VEGF), **(b)** angiostatin, **(c)** endostatin. Data are expressed as means ± SEM of 8 animals. ^#^ *P* ≤ 0.05 vs. normal control, *P ≤ 0.05 vs. colitis control.

Induction of colitis was associated with a distinct increase in the colonic levels of both angiogenic and antiangiogenic factors as compared to normal control. There were 6.5-fold increases in the levels of VEGF and endostatin as well as 4.5-fold increases in angiostatin levels. Niacin was effective in protecting against such rise in a dose-dependent manner (Fig. 4).

### Histological examination

**Figure 5:**
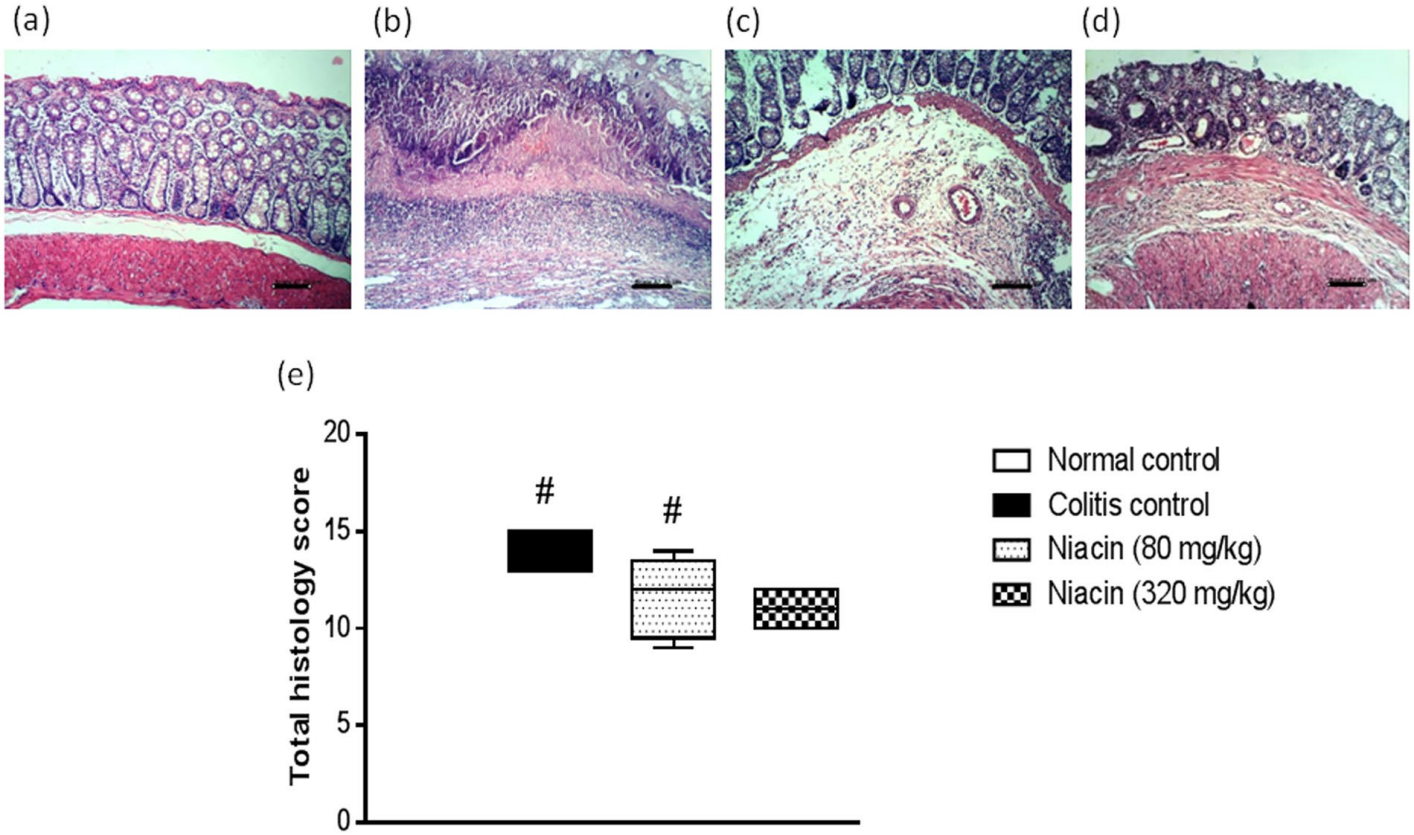
Effect of niacin on histopathological changes of rat colon in iodoacetamide model of colitis. (**a**) Normal control rat: normal histological structure of mucosa, (**b**) Colitis control rat showing necrosis of epithelium, inflammatory infiltrate in mucosa and submucosa as well as submucosal oedema, (**c**) Niacin (80 mg/kg) pretreated rat showing moderate inflammatory infiltrate in lamina propria and submucosa, **(d)** Rat pretreated with niacin (320 mg/kg) showing minimal changes. (H&E staining, ×100 original magnification). (**e**) Total histology score, data are expressed as box plots of the median of at least six animals. ^#^ *P* ≤ 0.05 vs. normal control.

Representative histological images of H&E-stained colon sections from each group are shown in Fig. 5. In contrast to normal control animals (Fig. 5a), iodoacetamide-treated animals (Fig. 5b) showed marked necrosis of the epithelium and submucosal edema. These changes were associated with massive inflammatory cell infiltration in the lamina propria and submucosa. The infiltrated inflammatory cells included neutrophils, lymphocytes, and macrophages. The total histology score was markedly increased in iodoacetamide-treated rats (Fig. 5e). Pretreatment with niacin tended to protect against the histological changes induced by iodoacetamide as evidenced by the lesser severity of the above parameters. The inflammatory infiltration in the mucosa and submucosa was only mild to moderate (Fig. 5c,d) and the total histology score was markedly decreased (Fig. 5e). The higher the dose of the drug, the greater was its protective effect.

### Role of GPR109A

**Figure 6:**
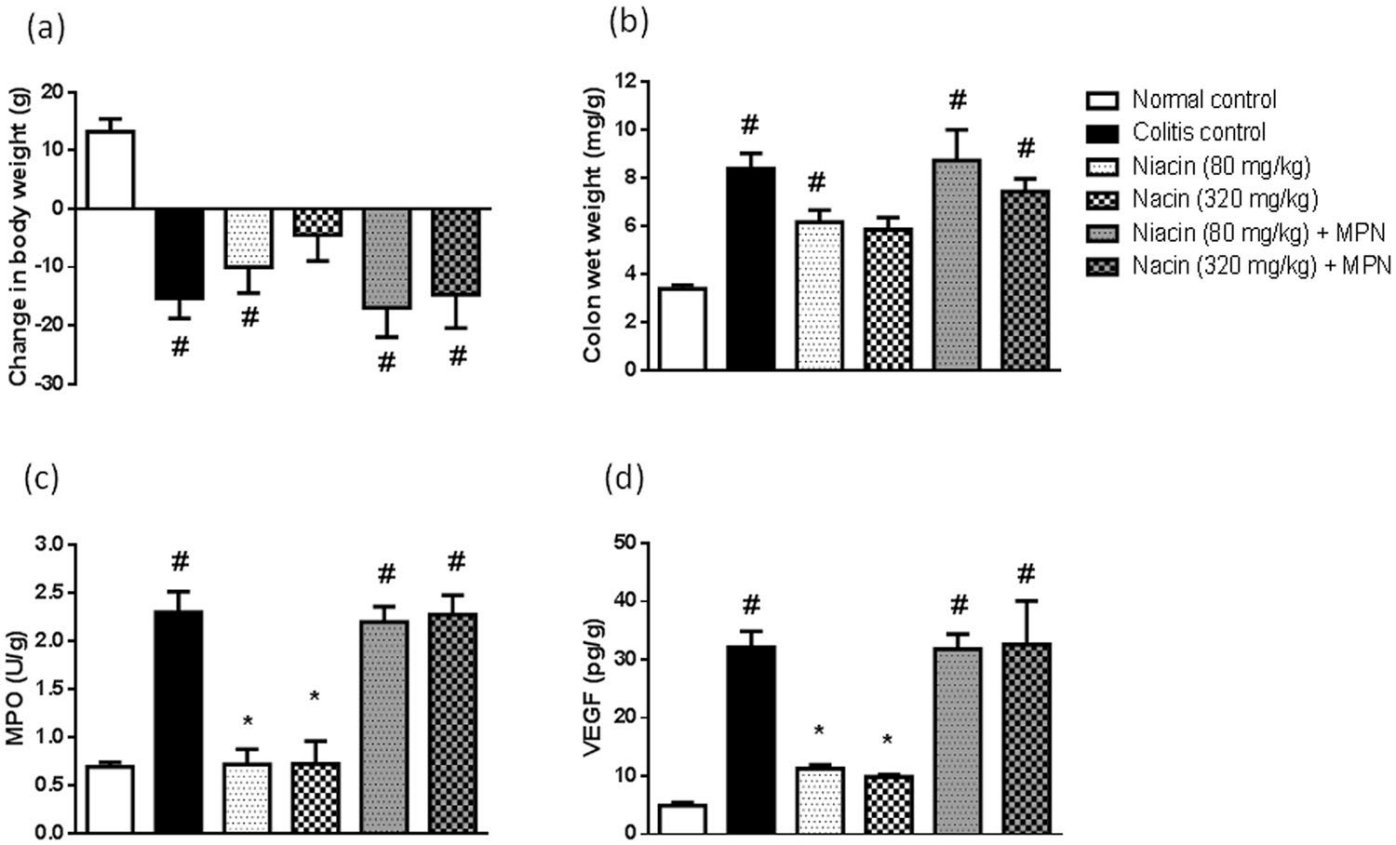
Role of GPR109A in the protective effect of niacin against iodoacetamide-induced colitis in rats. (**a**) increase in body weight of animals measured from the time of induction of colitis until sacrifice, **(b**) colon wet weight, (**c**) Myeloperoxidase (MPO) activity, (**d**) vascular endothelial growth factor (VEGF). Data are expressed as means ± SEM of 5 animals. ^#^ *P* ≤ 0.05 vs. normal control, *P ≤ 0.05 vs. colitis control.

In the presence of MPN, niacin failed to prevent iodoacetamide-induced loss in the body weight of animals. Moreover, MPN abolished the protective effect of niacin against iodoacetamide-induced rise in colon wet weight as well as the colonic levels of both MPO and VEGF (Fig. 6).

## Discussion

The current study revealed that niacin protected against experimental colitis induced by iodoacetamide in rats by ameliorating colonic inflammation and pathologic angiogenesis. This was demonstrated in the prevention of iodoacetamide-induced weight loss especially with the high dose level of niacin. Consistently, niacin was previously shown to ameliorate (AOM + DSS)-induced weight loss^12^. Improvement was also demonstrated in both macroscopic and microscopic indices of damage, where niacin pretreated rats showed marked decrease in colon wet weight and total histology score as compared to control colitis rats. The protective effect of niacin was reflected on the biochemical measurement. Niacin obviously lessened the colonic MPO activity in GPR109A-dependent manner. MPO activity, a hallmark of colonic inflammation, was up-regulated in colons of rats with colitis indicating massive leukocyte infiltration into the colon as verified by the histological examination. This increase in leukocyte infiltration is a characteristic feature of IBD and experimental colitis contributing to disease initiation and subsequent tissue damage^21, 22^. Infiltrated leukocytes produce cytokines (such as TNF-α), angiogenic growth factors (such as VEGF), proteolytic enzymes (such as matrix metalloproteinases; MMP-2 and -9), and oxidants^23–28^. Thus, it is very likely that leukocyte infiltration facilitates the inflammatory and angiogenic changes observed in IBD. Niacin, by decreasing colonic MPO activities, was expected to possess beneficial effects against both inflammatory responses and pathologic angiogenesis.

The anti-inflammatory activity of niacin was previously recorded in several *in vivo* and *in vitro* studies in which niacin was shown to decrease TNF-α expression and production via down-regulating nuclear factor (NF)-κB activation signaling pathway. The inhibitory effect of niacin on TNF-α production was found to be mediated by GPR109A^29–32^. Although several studies addressed the anti-inflammatory effect of niacin, only one study, to our knowledge, investigated this effect on colonic inflammation^12^. This study, however, was done in experimental colon cancer model. In the current study, niacin was found, for the first time, to inhibit TNF-α production in iodoacetamide-induced colitis model. TNF-α plays a crucial role in the pathogenesis of IBD, most likely because it disrupts the epithelial barrier, induces apoptosis of the villous epithelial cells, and stimulates the secretion of chemokines from the intestinal epithelial cells^33^. It also activates the adaptive immune system of the bowel by recruiting and activating neutrophils and macrophages^34, 35^. Moreover, inflammatory signaling via TNF-α up-regulates VEGF^36, 37^, a fundamental regulator of angiogenesis^38, 39^. TNF-α also increased the expression of vascular endothelial growth factor receptor-2 and its co-receptor neuropilin-1 in human vascular endothelial cells^40^. These findings support the existence of a direct link between inflammation and angiogenesis in IBD. Treating IBD patients with anti-TNF-α monoclonal antibody, infliximab, showed a rapid and sustained reduction in serum levels of VEGF^41^. Therefore, we expected that niacin, by reducing TNF-α and MPO, could affect angiogenesis that represents a critical component in IBD pathogenesis.

Clinical studies and animal models of experimental colitis showed increased microvascular density in the mucosal and submucosal tissue^15, 16, 42^ and up-regulation of VEGF^43–48^. Moreover, there was a strong causal association between increased VEGF expression and progression of experimental colitis. VEGF mRNA and protein expressions were increased as early as 0.5 hour after iodoacetamide enema and remained elevated in the active phase of colitis^48^. Consistently, the present findings revealed that colonic level of VEGF was markedly elevated in rats with idoacetamide-induced colitis. Up-regulated VEGF increases the expression of adhesion molecules, accelerates inflammatory cells adhesions^49, 50^, increases vascular permeability in colonic mucosa; thus, it facilitates inflammatory cell infiltration at the site of injury^38, 42, 48^. The infiltrated inflammatory cells up-regulated VEGF mRNA expression and increased VEGF protein levels. There was strong positive staining for VEGF in leukocytes in inflamed colonic tissue, whereas in normal tissue, VEGF was mostly localized to the endothelial cells^48^. Activated monocytes and/or macrophages alone are sufficient to induce angiogenesis^51^. This observed association between VEGF production and leukocytic infiltration in inflamed colonic tissues was consistent with the present findings, where colitis induction resulted in increased VEGF level and MPO activity indicating pathologic angiogenesis and increased neutrophilic infiltration. Neutralization of VEGF by anti-VEGF antibody was found to reduce leukocyte infiltration, inhibit angiogenesis and ameliorate colitis^48^. Consistently, pretreatment with niacin prevented the iodoacetamide-induced rise in VEGF level as well as MPO activity, an effect that was mediated through its anti-inflammatory properties. The ability of niacin to reduce VEGF was previously recorded when supplemented along with tamoxifen in breast cancer patients^52^. Butyrate was also found to repress angiogenesis *in vitro* and *in vivo* and reduce expression of proangiogenesis factors, including VEGF^53–56^. Moreover, Gambhir *et al*.^57^ revealed that the anti-inflammatory receptor GPR109A regulated the pathologic angiogenesis in diabetic retina. Up-regulation of GPR109A was associated with decreased expression of angiopoietin-like-4 (ANGPTL4), a gene that has received much attention as a critical regulator of pathologic angiogenesis and vascular permeability in retina. Additionally, absence of GPR109A (GPR109A^−/−^) was associated with upregulation of ANGPTL4. This association between GPR109A and regulation of pathologic angiogeniesis was also confirmed in the present study, where niacin’s protective effect against iodoacetamide-induced elevation in VEGF was abolished in the presence of MPN, an inhibitor of GPR109A. Niacin did not alter the colonic levels of VEGF in colitic rats treated with both niacin and MPN suggesting the essential role of GPR109A in mediating the niacin’s antiangiogenic properties in iodoacetamide model of colitis.

Angiogenesis is governed by a balance between pro- and antiangiogenic factors^58^. In the present study, we found that both angiogenic factor (VEGF) and antiangiogenic factors (endostatin and angiostatin) were significantly increased in the rat colon with experimental colitis. This was in agreement with the previous studies which showed concomitant upregulation of VEGF and anti-angiogenic factors endostatin and/or angiostatin in both rat and mouse models of colitis^47, 59, 60^. Moreover, Tolstanova *et al*. found a positive correlation between the levels of endostatin or VEGF and the sizes of colonic lesions in iodoacetamide-induced colitis^60^. The authors considered this concomitant increase in endostatin level to be a defensive response to the increased VEGF in colitis. Since niacin reduced the elevated VEGF, niacin was expected to protect against the rise in the anti-angiogenic factors in experimental colitis. In fact, the increased levels of VEGF, endostatin and angiostatin were reversed significantly by niacin in a dose-dependent manner. Previous study of Deng *et al*. demonstrated that mesalamine decreased endostatin and angiostatin as a result of reduced TNF-α expression that restore the balance between MMP2 and MMP9 in iodoacetamide-induced colitis model^59^. It is relevant to a clinical study that showed that therapy with infliximab increases MMP2 and decreases MMP9 in patients with Crohn’s disease^61^. Therefore, the ability of niacin pretreatment to reduce endostatin and angiostatin levels could be as a result of its anti-inflammatory activity and decrease in TNF-α as well as concomitantly to a reduction in VEGF levels.

Because GPR109A regulated the expression of the anti-inflammatory cytokine IL-10^12^, it is of interest to examine the effect of niacin on IL-10 production in colonic inflammation. IL-10 deficiency leads to spontaneous colitis^62–64^. Polymorphisms in the genes that encode IL-10 or IL-10 receptor are linked to increased incidence of IBD^65, 66^. Conflicting reports have been published on the effect of experimental colitis on IL-10 levels. In some studies a rise of IL-10 was observed^67–69^, while others showed no significant change in its levels^70^. The present findings revealed that the intra-colonic administration of iodoacetamide resulted in a decrease of colon levels of IL-10. This reduction in IL-10 levels was previously observed in several studies^71–73^. Pretreatment with niacin normalize IL-10 level in the colon of rats with iodoacetamide-induced colitis. This was consistent with the previous study of Singh *et al*. who showed that both butyrate and niacin induced the expression of IL-10 by splenic dendritic cells and macrophages^12^.

In conclusion, the present study revealed that niacin protected against colitis through its anti-inflammatory and anti-angiogenic effects in a GPR109A-dependent manner. These findings could have important implications for prevention as well as treatment of IBD and suggest that under conditions of reduced dietary fiber intake and/or decreased butyrate production in colon, pharmacological doses of niacin might be effective to protect colon against inflammation and pathogenic angiogenesis.
